# What helps hospital staff in times of crisis: qualitative results of a survey on psychosocial resources and stressors in German hospitals during the COVID-19 pandemic

**DOI:** 10.3389/fpubh.2023.1260079

**Published:** 2023-10-05

**Authors:** Kira Schmidt-Stiedenroth, Lisa Guthardt, Melanie Genrich, Mara Köhne, Maja Stiawa, Rebecca Erschens, Florian Junne, Imad Maatouk, The SEEGEN-Consortium, Harald Gündel, Peter Angerer, Andreas Müller

**Affiliations:** ^1^Institute of Occupational, Social and Environmental Medicine, Medical Faculty and University Hospital, Heinrich-Heine University Düsseldorf, Düsseldorf, Germany; ^2^Institute of Psychology, Work and Organizational Psychology, University of Duisburg-Essen, Duisburg, Germany; ^3^Department of Psychiatry and Psychotherapy II, Ulm University, Ulm, Germany; ^4^Department of Psychosomatic Medicine and Psychotherapy, University Hospital Tübingen, University of Tübingen, Tübingen, Germany; ^5^University Hospital for Psychosomatic Medicine and Psychotherapy, University Hospital Magdeburg, Magdeburg, Germany; ^6^Department of General Internal and Psychosomatic Medicine, University Hospital Heidelberg, Heidelberg, Germany; ^7^Department of Internal Medicine II, University Hospital Würzburg, Würzburg, Germany; ^8^Department of Psychosomatic Medicine and Psychotherapy, University Hospital Ulm, Ulm, Germany

**Keywords:** COVID-19, resources, psychosocial working conditions, hospital, Germany, stressors

## Abstract

**Background:**

Even before the COVID-19 pandemic, hospital workers faced a tremendous workload. The pandemic led to different and additional strain that negatively affected the well-being of employees. This study aims to explore psychosocial resources and strategies that were used by hospital staff.

**Methods:**

In the context of an intervention study, employees of three German hospitals were questioned in writing in summer and fall 2020. Five open-ended questions about the pandemic were asked to capture corresponding effects on daily work routine. Answers of 303 participants were evaluated using structuring qualitative content analysis.

**Results:**

Significant stressors and resources were identified in the areas of work content and task, social relations at work, organization of work, work environment and individual aspects. Stressors included, for example, emotional demands, conflicts, an increased workload, time and performance pressure. Important resources mentioned were, among others, the exchange with colleagues and mutual support. Sound information exchange, clear processes and guidelines and a positive work atmosphere were also important. In addition, the private environment and a positive mindset were perceived as helpful.

**Conclusion:**

This study contributes to a differentiated understanding of existing psychosocial resources of hospital staff in times of crisis. Identifying and strengthening these resources could reduce stress and improve well-being, making hospital staff better prepared for both normal operations and further crisis situations.

## 1. Introduction

Suboptimal working conditions in hospitals, such as staff shortages or non-transparent work processes, can negatively affect employees’ health and pose a risk to patient care (1–3). The pandemic led to additional demands, like an increased workload, more frequent interruptions or the pressure to increase test capacities, which negatively influenced the well-being of employees (4–7). However, workplace-related causes for stress were present in German hospitals even before the COVID-19 pandemic. These included increasing treatment figures and excessive workload at times (2, 8) and were attributed to political changes in the healthcare system and a related limited scope for improving working conditions (9).

This already critical situation in German hospitals was exacerbated by the COVID-19 pandemic. Several studies addressed pandemic-specific stressors experienced in hospitals and/or the pandemic’s consequences on the mental health of employees in the healthcare sector (4–7, 10–17). It is therefore evident that stress prevention in hospitals is essential (18, 19).

Resources become especially important in times of crisis (20). Empirical studies have indicated that stressful working conditions can be better managed when strong resources are available (20). Occupational psychology developed different theoretical models to explain the connection between various work demands, resources and stress. One of these is the “job-demands-resources-model” (JD-R theory) by Bakker, Demerouti et al., which hypothesizes that job strain results from an imbalance between the demands that employees are exposed to and the resources available to them (21–23). Demands refer to “all physical, psychological, social or organizational aspects of the job that require sustained physical and/or psychological (cognitive or emotional) effort or skills and are therefore associated with certain psychological and/or physiological costs” (21). Demands are not necessarily negative. However, they can become stressors if they are combined with a high level of effort from which employees cannot recover (21). In this model, resources are defined as the physical, mental, social or organizational aspects of work that serve the achievement of work goals, reduce work demands and promote individual growth, learning and development (21). Yet, individual resources such as self-efficacy and optimism can also play a similar role as work resources (22). After the COVID-19 pandemic, the authors updated the JD-R theory by also including home and personal demands and resources, proposing that these interact with organizational and job demands and resources (24).

Compared to the large amount of literature on stressors during the COVID-19 pandemic, there are overall mainly quantitative studies on psychosocial resources of hospital staff during this crisis (4, 7, 10). These studies report, among other things, that the resilience of nursing staff was largely influenced by working conditions (4) and that family, friends and leisure time were also important resources among hospital workers (7). Caregivers who also worked outside the hospital assessed the key resource of interpersonal relations most positively (25, 26). Qualitative studies can be useful to fully understand quantitative data, or to provide further insights we may not know that are missing from quantitative studies (27). To our knowledge, qualitative approaches concerning psychosocial resources of hospital staff during the COVID-19 pandemic have received less attention in the research literature. An interview study from the United States examining coping strategies reported that healthcare workers and first responders managed to better cope with the crisis by gathering information and strategies, by seeking support and by practicing self-care (28). Another qualitative study from Italy reported that individual adaptability and engagement, mutual support and teamwork, leaders’ support as well as information and communication technologies and personal protective equipment, among others, were perceived as job resources by healthcare workers (29). Both qualitative studies were concerned with the first wave of the pandemic in their respective countries.

The present study, in which hospital workers from different occupational areas were questioned during the first two waves of the pandemic in Germany, is meant to capture individual impressions and perspectives in order to deepen existing knowledge on the topic by adding the second pandemic wave to the few existing qualitative studies on resources. The aim of this study is to identify specific resources that were mentioned as helpful in the context of stress during the COVID-19 pandemic in Germany and that may not have been captured in previous studies on the topic. Exceptional situations can be useful in order to learn for both crises and day-to-day work. The qualitative method allows us to explore subjective assessments of hospital staff regarding helpful resources and strategies as well as stressors during the COVID-19 pandemic. Studies indicate that reinforcing existing resources of employees can have a positive impact on the handling of stressors in general and on the overall working situation (30). Therefore, we address the following research question: What psychosocial resources and strategies were useful for hospital staff in order to counteract stressors faced during the COVID-19 pandemic?

## 2. Methods

### 2.1. Data collection and study participants

The present study was conducted as part of the collaborative project “SEEGEN–Mental Health at the Workplace Hospital,” which was implemented from 2017 to 2022 at three hospital sites of different sponsorship in Germany (8). Of the three participating hospitals, one was a university hospital, one was a community hospital and the third was a hospital owned by a private company. The aim of the research association was to develop and evaluate a complex intervention for health promotion at the hospital workplace. In this context, written surveys at three different times of measurement were planned. Detailed information on the SEEGEN study design has already been published (8).

The SEEGEN study was registered in the German Clinical Trials Register (DRKS) under the DRKS-ID DRKS00017249. Positive votes from the ethics committees involved were obtained (University of Ulm: 501/18, University of Heidelberg: S-602/2019, University of Düsseldorf: 6193R). Inclusion criteria for the study were age between 18 and 70 years, written informed consent and sufficient knowledge of the German language. All employees of the three hospitals in the 18 cluster units (6 clusters per hospital) being involved in patient care and meeting the inclusion criteria were eligible to participate in the SEEGEN study. Baseline recruitment took place from October 2019 until March 2020 through information events at each site. Within the cluster-randomized trial, *N* = 5,654 individuals were eligible to participate. After 466 participants had been recruited, 407 persons took part in the baseline survey at the end of 2019, which represents a response rate of 88.1%.

**Figure 1:**
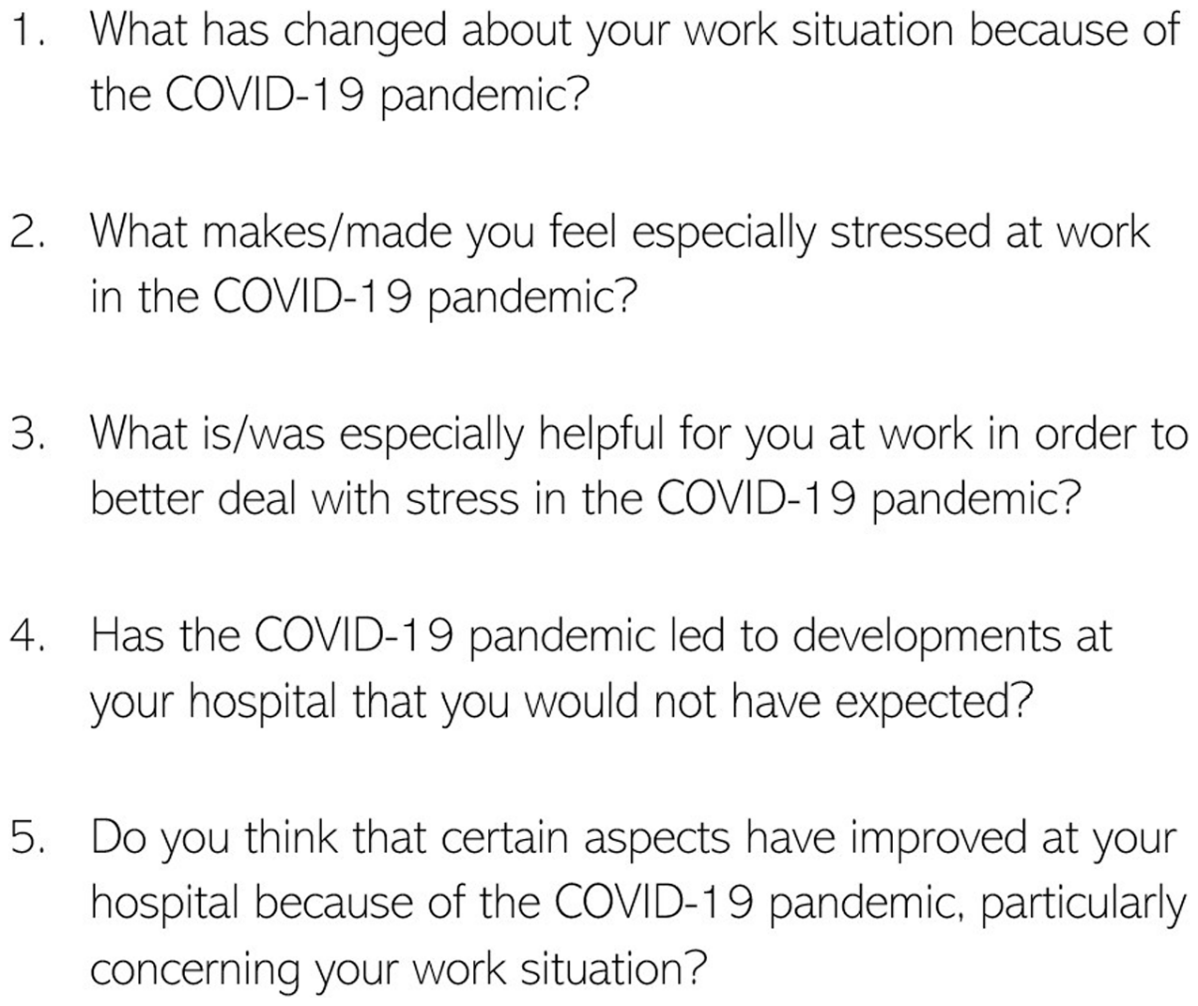
Items/open questions of the written follow-up surveys.

After the outbreak of the COVID-19 pandemic, five open-ended questions were developed ad hoc and posed in the two planned written follow-up surveys (T1 and T2) to assess the impact of the pandemic on the participants’ work routine (Figure 1). The present study is based on the answers to these questions, posed only in T1 and T2. At that time, information on the pandemic and its related impact was still scarce, hence the added open-ended questions were kept simple in order to be able to cover a broad range of possible impacts and changes. The inclusion of open-ended questions in surveys is considered a pragmatic approach to obtain deeper insights into complex questions in a timely manner (31). The first follow-up survey (T1) took place in summer 2020 and followed phase 1 of the pandemic in Germany, which was characterized by the novelty of the disease, an increase in infections and the non-availability of uniform procedures and guidelines (32). The second follow-up survey (T2) took place in fall 2020, which came after the so-called summer plateau phase characterized by milder infections (32). A total of 317 and 267 persons took part in the T1 and T2 surveys, respectively. Out of these, 303 employees answered at least one of the questions in one of the two written follow-up surveys. Of these 303 individuals, 173 participated in both T1 and T2, and 130 participated in either T1 or T2 only.

Due to the fact that the five open-ended questions concerning the pandemic were embedded into the SEEGEN survey, there was no separate sampling for potential participants of our study. As data were collected through a written survey, no relationship was established between the researchers and the participants. Further, the written survey format did not allow us to ask participants more in-depth or comprehensive questions. For this reason, thematic saturation could not be strived for (see limitations). After completion of the SEEGEN project, results were reported to participants. However, these were limited to the complex intervention and did not include the results of the present study.

### 2.2. Data analysis

The pandemic was characterized by highly dynamic situations and at times rapid developments. In order to represent a preferably large spectrum of hospital working conditions during the COVID-19 pandemic, both time points were analyzed together. Yet, we indicated significant differences between both points in our analysis.

The qualitative content analysis of the five open-ended questions was conducted through manual coding by an interdisciplinary team of researchers, which is an established method for the qualitative analysis of open-ended survey questions (31, 33). Four of the authors (KS, LG, MG and MK) were involved in the coding process in order to reduce researcher bias. At the time of data analysis, these four authors had different levels of experience in qualitative research analysis as well as academic backgrounds: PhD in Medical Anthropology (KS), bachelor’s degree in English Studies and master’s degree in Literary Translation (LG), Diploma in Educational Sciences (MG), undergraduate student of Work and Organizational Psychology (MK). This variety facilitated a profound and diversified analysis of the data. KS, LG and MG were employed as academic staff in the context of the SEEGEN project, while MK was a student research assistant (see affiliations). In order to further avoid bias, the manuscript was revised and commented by the remaining authors, who represent various genders and backgrounds (mainly psychology and medicine) and some of whom work as hospital staff themselves.

The coding process was divided into four steps. In the first step, the answers of the paper questionnaires were digitalized and fed as documents in the MAXQDA software together with the already digitalized data from the online surveys. The analysis of the open-ended questions via qualitative content analysis following Kuckartz (34) was conducted by MG and MK in a multistage procedure using the MAXQDA software. We used deductive categories derived from central features of work design according to the recommendations of the Joint German Occupational Safety and Health Strategy (GDA; Beck et al. 22. November 2017) and inductive categories formed from the data to build categories. Following this methodology, we established definitions, examples from the material as well as coding rules and reviewed and slightly adjusted the code system based on a sample of 50 transcriptions. In the second step, KS and LG performed an additional quality check of the coded material using MAXQDA 2020. The code system was further developed by going through the material two more times and by further adjusting existing categories in an iterative process using consensual coding (34). During this stage of the coding process, KS and LG met once a week and discussed categories and codes considered insufficient, missing or misclassified, deciding through consensus. In this process, the category system was further augmented with inductive dimensions that emerged from the data and were not represented by the features of the GDA. Discussions and changes in the category system from the code meetings were recorded in minutes.

Our code system operated on three levels: First level (codes), second level (sub-codes) and third level (characteristics). The first two levels addressed the thematic domain (e.g., organization of work (code) and work time (sub-code)), whereas the third level categorized these contents as positive, negative or neutral. Through the third level, we aimed at identifying possible resources (positive characteristics) and stressors (negative characteristics) and to omit statements from our analysis that could not be clearly classified in either way (neutral characteristics). The resulting code system for the first two levels is shown in Table 1. We aimed at a high quality of coding by consensual coding and verified it through intercoder reliability (34). Both KS and LG coded a sample of 50 documents (25 from each T1 and T2) according to the developed code system. The function of the intercoder agreement in the MAXQDA software yielded suitable values of matching coding (74% for T1 and 86% T2). We also added demographic data of the participants that had been collected in the baseline survey to the dataset.

**Table 1:** Code system.

Codes and sub-codes	Definitions/rules of application	Examples from the material
1 Work content and task		
1.1 Scope of action	Influence on work content, workload, work methods/procedures, sequence of the tasks	“No free choice of patients possible anymore.” (T1, 64, Pos. 1) under changes
1.2 Variability/rich variety	Variety of requirements in terms of work equipment/objects and actions	“New remits.” (T2, 91, Pos. 5) under improvements
1.3 Responsibility	Competencies and responsibilities that are not related to changing guidelines, rules or work processes	“Transfer of responsibility in competence areas.” (T1, 105, Pos. 4) under unexpected developments; “Always consultation with doctor, not always clear decisions.” (T2, 151, Pos. 2) under stressors
1.4 Qualification	Changed qualification requirements of employees; new instruction/initial training, changed possibilities for further training	“More and more employees do not feel up to their task or are not sufficiently supported.” (T2, 144, Pos. 4) under unexpected developments; “Knowledge growth strongly increased.” (T1, 37, Pos. 1) under changes
1.5 Emotional demands	Experiencing emotionally touching events; clear reference to emotions and own needs. Emotion regulation required or not. Emotional demands in relation to work activities, in contact with patients or relatives. Not in relation to colleagues/supervisors -> social relations, supervisors, colleagues	“Patients are more aggressive overall due to waiting times at the door or also because facial expression is covered.” (T1, 15, Pos. 1) under changes; “More anxious and psychologically impaired patients.” (T2, 163, Pos. 2) under stressors
2 Organization of work		
2.1 Work time	Change in work time, other shifts or night work, change in overtime, breaks and in work on call	“As a result, many overtime hours could be reduced.” (T1, 110, Pos. 5) under improvements
2.2 Amount of work	Change in time pressure/work intensity, disturbances/interruptions, changed clocking	“Due to the COVID-19 pandemic workload was lower.” (T1, 63, Pos. 1) under changes; “A lot of delay due to the very strict hygiene regulation.” (T2, 23, Pos. 1) under changes; “Balancing act between patient care and the large number of organizational tasks.” (T1, 74, Pos. 2) under stressors
2.3 Work process	New, changed work processes, clear or unclear rules and/or guidelines	“Clear structures and requirements from hygiene and senior management.” (T1, 1, Pos. 3) under resources; “Clear rules from the employer.” (T2, 159, Pos. 3) under resources; “Frequent changing of procedures – feels like 1,000 e-mails per day.” (T1, 15, Pos. 2) under stressors; “Confused and changing guidelines from the employer.” (T2, 191, Pos. 2) under stressors
2.4 Information/communication	Change in the provision of information, changed processing/presentation of information, changing information	“Good information from the hygiene department, exchange with other employees.” (T2, 66, Pos. 3) under resources; “Clear information about the current situation in the clinic.” (T1, 23, Pos. 3) under resources; “No good possibility to get information anymore, no exchange.” (T2, 60, Pos. 4) under unexpected developments; “Lack of communication about changed processes.” (T1, 86, Pos. 2) under stressors
2.5 Cooperation	Changed commitment to the workplace, different opportunities for cooperation/support, different areas of responsibility	“Yes, support from areas that had reduced their workload.” (T1, 32, Pos. 4) under unexpected developments; “Little contact to other wards and colleagues, no networking.” (T2, 60, Pos. 1) under changes
2.6 Staff and work planning	Staffing, work planning including sick leave, vacation, quarantine, compensation for overtime	“Sufficient staff.” (T1, 115, Pos. 3) under resources; “Quarantine of colleagues burdens the working schedule.” (T2, 21, Pos. 1) under changes; “Staff shortage.” (T1, 136, Pos. 2) under stressors
3 Social relations		
3.1 Supervisors	Reference to feedback, recognition, support or appreciation	“Ward manager took our concerns seriously.” (T1, 56, Pos. 3) under resources
3.2 Colleagues	Reference to social contacts with colleagues, harmony, support and appreciation by colleagues	“Good support among each other.” (T2, 32, Pos. 3) under resources; “Exchange with colleagues.” (T1, 26, Pos. 3) under resources; “Mood of colleagues is generally worse, everyone is annoyed.” (T2, 20, Pos. 2) under stressors; “Lack of understanding among some colleagues in relation to necessary measures, constant discussions on this topic.” (T1, 183, Pos. 2) under stressors
3.3 Patients/relatives	Social interactions with patients and relatives, reference to recognition/appreciation	“Relatives are potentially more polite and more likely to agree with measures related to patients.” (T2, 69, Pos. 4) under unexpected developments; “Distance to patients.” (T1, 57, Pos. 2) under stressors
4 Work environment		
4.1 Physical and chemical factors	Reference to noise, lighting, hazardous substances, risk of infection, hygiene	“Hygiene measures.” (T2, 11, Pos. 5) under improvements; “Working in unsuitable rooms without air exchange with FFP2 mask.” (T2, 76, Pos. 1) under changes
4.2 Corporeal factors	Changed ergonomic design; different physical work, changed strain due to protective measures	“By wearing protective clothing. This is very stressful, especially physically.” (T2, 3, Pos. 2) under stressors
4.3 Workplace and information design	Changed workspace/patient rooms; changed design of signals and notes, changed visiting regulations	“The visiting ban made the corridors and patient rooms significantly emptier. Life was more relaxed for patients because there were very strict visiting hours, which made resting phases possible. As a caregiver you felt less harassed and threatened.” (T2, 21, Pos. 4) under unexpected developments
4.4 Work equipment	Reference to tools and work equipment; changed operation or setup of machines; use of software and protective clothing	“Not enough FFP2 masks, or very spare distribution of protective masks.” (T2, 21, Pos. 2) under stressors
4.5 Work atmosphere	Reference to mood and atmosphere at work, sense of community or no sense of community, cohesion within the team. This category does not refer to single factors only (e.g., social relations), but covers the entire social dimension.	“Good work atmosphere, good team and new, nice colleagues.” (T2, 77, Pos. 3) under resources
5 Individual changes/stressors/resources/strategies	Individual situation, approaches or private conditions	“Conversations with family and friends.” (T1, 86, Pos. 3) under resources; “Leisure time, e.g., hiking, cycling.” (T1, 97, Pos. 3) under resources; “Strain-bearing capacity is still exhausted.” (T2, 14, Pos. 1) under changes
6 Other changes	Other changes, improvements or deteriorations (stressors, resources)	“Medicine is more paramount than purely economic considerations.” (T1, 37, Pos. 5) under improvements; “It’s all about COVID at moment, but there are enough other diseases that have priority but are neglected.” (T2, 156, Pos. 2) under stressors
7 Question-oriented codes		
7.1 Changes	Concrete reference to changes according to the question	“More time-consuming patient handling due to corona tests and questioning.” (T2, 5, Pos. 1) under changes
7.2 Stressors	Concrete reference to stressors according to the question	“Distance to fellow people, patients, colleagues. Changed processes with severe limitations. Both during work time and during breaks.” (T2, 22, Pos. 2) under stressors
7.3 Resources	Concrete reference to resources according to the question	“Walks in the forest.” (T2, 4, Pos. 3) under resources
7.4 Unexpected developments	Concrete reference to unexpected developments according to the question	“Yes, mental load is indeed very high for many, and the resulting dissatisfaction is getting higher and higher. Some of the employees start making each other look negative, and the potential for dispute increases.” (T2, 38, Pos. 4) under unexpected developments
7.5 Improvements	Concrete reference to improvements according to the question	“It has become a bit more quiet (fewer patients, no visitors).” (T2, 8, Pos. 5) under improvements
7.6 Suggestions/wishes for future improvements	Concrete expression of wishes or suggestions. Improvements must not have been implemented yet.	“The appreciation of care staff has hopefully gotten better in the long run.” (T1, 33, Pos. 5) under improvements

In the third step, we conducted exploratory analyses to identify anomalies in the code and sub-code frequencies as well as important contents and possible patterns related to demographic characteristics. For this purpose, we conducted frequency analyses using the crosstabs function in MAXQDA. We further identified the most frequent resources, stressors and neutral demands. In the fourth step, we created content summaries of the most frequent stressors and resources.

## 3. Results

The sociodemographic characteristics of the study participants, collected in the baseline survey, are shown in Table 2. A total of 303 participants answered at least one of the open-ended questions in the T1 and T2 surveys, with an overlapping of 173 participants who participated in both surveys. We considered all answers as stand-alone, regardless of the survey time. Thus, no comparisons regarding changes across time were made. Many stressors and resources were present at both survey time points. Nevertheless, there were fluctuation patterns that can be explained by the temporal development of the pandemic and thus by changes in the work routine at the hospital. We have indicated cases where these patterns differed between survey time points.

**Table 2:** Description of participants’ sociodemographic characteristics (collected in the baseline survey for all study participants).

	Participated in T1 (*N* = 259)	Participated in T2 (*N* = 217)
**Age groups in years**		
< 21	0	0
21–30	36	29
31–40	49	41
41–50	59	48
> 50	96	81
No response	21	18
**Gender**		
Female	183	158
Male and divers	71	58
No response	5	1
**Occupational group**		
Medical service (medical service and medical-technical service)	63	50
Care service	113	97
Functional services (functional service, secretariats and others)	62	53
No response	21	17
**Management responsibility**		
With management responsibility	98	77
Without management responsibility	144	123
No response	17	17

In order to illustrate the background of stress, we will first present the most frequently mentioned stressors before addressing the resources. For a better overview, we summarized the most important results under the following categories: (1) work content and task, (2) social relations at work, (3) organization of work, (4) work environment and (5) individual stressors or resources. Tables 3, 4 provide specific exemplary quotes for the stressors and resources sorted by code. All verbatim quotes were translated from the original German by LG.

**Table 3:** Exemplary quotes concerning stressors (negative characteristics), sorted by codes.

Codes	Quotes
Work content and task	“That patients could not be visited, and doctors were not sensitive there.” (T1, 56, Pos. 2)
	“The visiting ban for relatives makes care more difficult because it’s a great burden for the psyche of patients and relatives.” (T2, 21, Pos. 1)
	“Tension between COVID-rules and personal freedom of patients. Sometimes, rules that contradict therapeutic recommendations need to be advocated.” (T2, 166, Pos. 2)
	“Patients are also insecure – they expect care staff to provide information.” (T1, 161, Pos. 2)
	“Constantly reminding patients and visitors to adhere to protective measures.” (T1, 220, Pos. 2)
	“Especially relatives/visitors insult care staff more severely.” (T2, 55, Pos. 1)
	“It was humanly and ethically not okay to let people die alone without any relatives, or not to pray together.” (T1, 154, Pos. 1)
	“Need for triage with regard to scarce surgery capacities.” (T1, 62, Pos. 2)
Social relations	“For one thing, the ignorance of some colleagues, so that you had to justify why you wanted to keep minimum distances. You felt more like the odd one out if you wanted to adhere to the hygiene and infection control measures within the team as well.” (T2, 21, Pos. 2)
	“Additional safety measures that colleagues and employees do not adhere to because they feel immune.” (T1, 91, Pos. 2)
	“Less collaboration, hardly any joint social events possible, e.g., eating cake to celebrate birthdays, having coffee or lunch breaks together.” (T2, 288, Pos. 2)
	“No personal support from supervisors, if communication takes place, it’s only pressure.” (T1, 50, Pos. 2)
	“Staff shortage exacerbates pressure on supervisors to keep the numbers up despite the pandemic.” (T2, 232, Pos. 1)
	“Desire from the management level to renounce to a lot in your personal life to maintain work capacity.” (T2, 256, Pos. 2)
	“Many discussions with patients concerning scheduling.” (T1, 14, Pos. 1)
	“The personal contact to your patients -> has become more impersonal (mouthguard, gloves, protective gown...).” (T1, 165, Pos. 1)
Organization of work	“Too many changes that have to be implemented in a short period of time lead to more work and overtime.” (T2, 155, Pos. 1)
	“Same number of persons, less time.” (T1, 46, Pos. 1)
	“Usually no breaks possible.” (T2, 172, Pos. 2)
	“After capacity had been booted up again, patients were significantly sicker + more labor-intensive.” (T1, 251, Pos. 1)
	“Always available, boundary between work and private time becomes blurred.” (T2, 281, Pos. 1)
	“Due to shortfall of personnel, number of staff is not sufficient to be able to adequately deal with the organization of tasks.” (T2, 284, Pos. 2)
	“Disinformation from the employer about planned measures, processes. Lack of communication.” (T1, 142, Pos. 2)
	“Dissatisfaction in the team due to changing orders.” (T1, 155, Pos. 4)
Work environment	“Working with full protective equipment is very exhausting; talking, breathing is very burdensome and also sweating.” (T2, 154, Pos. 2)
	“That protective material FFP2 masks + gowns were missing or should be re-used at times.” (T1, 48, Pos. 2)
	“No consistent procedures for isolation measures. No swab tests were carried out for suspected COVID-cases.” (T1, 29, Pos. 2)
	“No separate protection for employees and risk patients.” (T2, 233, Pos. 2)
	“Small meeting rooms, few possibilities to keep minimum distances.” (T2, 75, Pos. 2)
	“Potential risk of infection (for employees and oneself) due to poor screening (e.g., lack of tests in the emergency room).” (T2, 88, Pos. 2)
	“Higher burden to work in personal protective equipment and also to organize it.” (T2, 32, Pos. 1)
	“The team in the corona ward is emotionally exhausted.” (T2, 73, Pos. 4)
Individual stressors	“Fear of infecting myself and then especially my relatives.” (T2, 129, Pos. 2)
	“Great insecurity and fear among employees.” (T2, 57, Pos. 2)
	“Uncertain future.” (T1, 80, Pos. 2)
	“Loneliness.” (T1, 191, Pos. 2)
	“Furthermore, private compensation through positive activities is missing.” (T2, 152, Pos. 2)
	“In the private surroundings, many have backed away because you are working at the hospital.” (T1, 151, Pos. 2)
	“You always have to be a role model for everyone, more than usual because everyone is more observant.” (T1, 64, Pos. 2)
	“The fear of not making the right decision.” (T2, 239, Pos. 2)

**Table 4:** Exemplary quotes concerning resources (positive characteristics), sorted by codes.

Codes	Quotes
Social relations	“The good relation to my supervisor, the cohesion with my colleagues from my department.” (T1, 14, Pos. 3)
	“[…] This open communication has had the effect that other colleagues have also admitted that they want to adhere to the distance regulation.” (T1, 21, Pos. 3)
	“Exchange with colleagues, doctors and at team meetings.” (T2, 17, Pos. 3)
	“We talk a lot with colleagues at work during breaks, respectively, the appreciation from supervisors has helped me. They motivated us and told us we would handle everything well. Sometimes, I was also proud that we had sticked together so well.” (T1, 154, Pos. 3)
	“Great team -> everyone helps everyone -> very good cohesion.” (T1, 279, Pos. 3)
	“More intensive cohesion among individual colleagues.” (T2, 288, Pos. 3)
	“The caring way colleagues treated each other. The cohesion.” (T2, 9, Pos. 3)
	“Conversations with colleagues, the exchange, their feelings/opinions.” (T2, 185, Pos. 3)
Organization of work	“By reducing patient occupancy while increasing the number of staff, there is less stress and time pressure.” (T1, 206, Pos. 1)
	“You have more time for the patients.” (T2, 54, Pos. 5)
	“Deceleration has been noticeably good.” (T1, 76, Pos. 3)
	“Fewer patients at times, thus relief, beneficial.” (T2, 215, Pos. 1)
	“Newsletter, information, regular meetings where the next steps were worked out.” (T1, 98, Pos. 3)
	“Timely information and clear process instructions.” (T2, 78, Pos. 3)
	“Clear instructions, not something different every day, enough staff to relieve everyone.” (T1, 160, Pos. 3)
	“Consultation with management and other departments affected, e.g., hygiene, purchasing.” (T2, 155, Pos. 3)
Work environment	“In my opinion, there are less infects also among patients due to fewer visitors in the clinic/in the patient room. The clinic is calmer, work is therefore not so stressful. The noise level is lower.” (T2, 221, Pos. 5)
	“The introduced visiting hours are pleasant. This should be kept.” (T2, 220, Pos. 3)
	“Good collegial interaction throughout the hospital.” (T1, 204, Pos. 3)
	“Mastering tasks together, good agreements. Team spirit is enhanced, because things only work together.” (T1, 265, Pos. 3)
	“I mostly solved the problems myself with the help of the accessible (very good!!!) material and the support from our hygiene specialist. I felt challenged thus not burdened.” (T1, 286, Pos. 3)
	“Wearing protective equipment because of the burden of the fear of infection.” (T2, 124, Pos. 3)
	“Currently the availability of FFP2 masks, patients and accompanying persons are tested.” (T2, 222, Pos. 3)
	“Increasing acceptance of the need for protective measures within the team, more protective equipment.” (T1, 21, Pos. 3)
Individual resources	“Good and strengthened private ‘environment’ -> I’m always looking forward to coming ‘home’.” (T1, 279, Pos. 3)
	“Activities in daily life. Doing something with friends and family.” (T1, 185, Pos. 3)
	“Strong social environment both with family and team.” (T1, 173, Pos. 3)
	“Outdoor sports, long walks.” (T2, 107, Pos. 3)
	“Relaxing in nature.” (T1, 283, Pos. 3)
	“I’m used to dealing with a lot of stress, I applied my compensation mechanism to the pandemic as well -> Hanging in there, showing optimism despite the difficult situation etc.” (T2, 74, Pos. 3)
	“Serenity and hoping for improvement.” (T2, 106, Pos. 3)
	“A positive way of thinking and a positive approach.” (T2, 95, Pos. 3)

### 3.1. Stressors

#### 3.1.1. Work content and task

Participants mainly discussed stressors related to emotional demands and ethical conflicts in this context. These stressors were mentioned more frequently by employees from the nursing service and the secretariats and in the second survey time point. Among these stressors we found psychological stress, increasing anxiety of patients, loneliness, physical distance and aggression/irritability also among patients’ relatives. Communication and general contact were perceived as more difficult due to the use of masks. Participants also reported ethical conflicts, e.g., in dealing with the deceased or with dying persons (missing relatives, not dignified, triage).

#### 3.1.2. Social relations at work

Disagreements and conflicts between colleagues, especially concerning distance and hygiene regulations, irritability, a lack of understanding and missing exchange or contact with colleagues were perceived as stressful. Employees also mentioned stressful conflicts with superiors (e.g., lack of support, more pressure, e.g., to renounce to certain aspects in the private environment for the sake of work). Participants also brought up stressors in the handling of patients and relatives (e.g., distance, limited interaction, discussions). The number of statements concerning stress caused by relations with colleagues and by contact with patients and relatives increased in the second survey period.

#### 3.1.3. Organization of work

Overall, participants reported an increase in workload and in the amount of work and in this context also more stress, excessive demands, time and performance pressure due to additional tasks and also because of absent colleagues (e.g., more administrative tasks, implementation of guidelines, make up for canceled appointments). Work time-related stressors came up more frequently in the second survey stage. Other issues mentioned in this context were staffing shortages becoming especially apparent due to illness or quarantine absences, for example. Respondents described constantly changing guidelines, regulations and procedures (sometimes daily changes) as a major burden in the workflow. Some of the guidelines were perceived as contradictory or unclear, which seemed to have led to uncertainties. Overall, many employees lacked clear communication, reliable information and clarification, especially in the initial survey stage. Stress also seemed to result from additional cooperation and necessary arrangements. In relation to the general communicational exchange, employees described a lack of networking and less productive communication due to a lack of meetings or online conferences.

#### 3.1.4. Work environment

Employees mentioned stress caused by wearing protective equipment (in some instances for a long time), especially mouth and nose protection (circulatory problems, breathing and skin problems, headaches), but also by poor quality, unsuitable protective clothing or the lack of protective equipment and tests. According to the employees, protective equipment sometimes had to be organized by themselves or one-time material had to be re-used. For the participants, an additional burden was present in the context of the perceived risk of infection (e.g., bad screening, lack of control, inconsequent implementation of protective measures, missing uniform procedures). Statements on stress caused by the work environment increased in the second survey stage.

#### 3.1.5. Individual stressors

Individual stressors covered fear of infection (fear of infecting family members, patients or fear of being infected) and a general uncertainty regarding the pandemic (e.g., uncertain future, unpredictable course of the pandemic, possible lockdowns and restrictions). Additionally, participants described individual pressure or stress (e.g., tension, high mental burden) and stressors such as panic, isolation or distrust. Some participants said they were more cautious and more distanced while others rather experienced this behavior from other people. According to the participants, further stressors emerged from the pressure of not wanting to make mistakes or trying to act as a role model and from social and economic changes (e.g., child care, extremization of society). This type of stress occurred more frequently in the first survey stage.

### 3.2. Resources

Table 5 illustrates resources that participants mentioned most frequently in relation to the pandemic. Only minor differences between the individual occupational groups occurred. Very few resources were described in the section of work content and task, which is why this part is omitted in the following.

**Table 5:** Thematic summary of the resources (positive characteristics) in relation to the COVID-19 pandemic, the 10 most frequent codes.

Main codes	Sub-codes	Specific resources
Social relations	Colleagues	Cohesion and support: Team spirit Exchange and common handling Team stability Social backing Colleagues: Good collaboration Good relations Open communication Positive behavior
Organization of work	Amount of work	Patients, relatives and capacity: Reduction of examinations, beds and operations Fewer elective cases and emergencies Fewer patients More time for individual patients Fewer relatives, fewer conversations needed Tasks and processes: Fewer tasks All tasks that had been left could be handled Better planning in advance possible Less time pressure Fewer team meetings Workload: Less work strain Less workload More calmness Deceleration Less stress
	Information/communication	Information as a resource: Newsletter Good/improved flow of information Exchange of information Exchange and communication in general: Improvement in communication Weekly meetings in the corona steering committee Regular meetings to plan further actions Daily information meetings News ways of communication (e.g., improvement of digital communication, information on the internet, daily updates via e-mail, virtual conferences and trainings) Communicative exchange Addressing problems Supervision Clarification Clarity/Transparency: More transparency and openness Transparent leadership team Information on COVID-19 and rules: Clear information on current situation Updates on COVID-19 as short videos Safety through education Timely information about measures and changes Media with new information Clear instructions
Organization of work	Work process	Structures and guidelines: Clear structures and specifications (from the hygiene, senior management, in the department and at the hospital, precise guidelines from the management) Fixed structures that were not constantly changed Faster and more pragmatic decisions Less confusion Restructuring Processes in general: Processes became slower and calmer Hectic is avoided Clear information about the current situation and processes Clarity Increasing routine Discuss results and conduct initial interviews by phone/video Confidence in own processes Planning and organization in general: Good organization Good considerations and rational division to avoid shortages Better prepared tasks Containment of the first phase by shutting down operations
	Cooperation	Collaboration: Support of other departments Helping out at other wards More constructive collaboration with the administration/the executive level Joint implementation strategies More intensive coordination with the clinic management Insights into other areas Good interaction among colleagues Formation of interprofessional teams Establishment of communication structures Cohesion and understanding: Better mutual understanding within and between departments Good cohesion Strong team to back each other up Exchange and support: Exchange with other areas, wards Solving problems with the help of the hygiene specialist Exchange with colleagues, doctors and at team meetings Support from hospital hygiene/hygiene officers Making arrangements
Work environment	Workplace and information design	Regulations for visitors/entrance: Selecting patients at the gate and directing them to the appropriate department Access controls Fewer visitors Regulated visiting hours Relatives partly recognized as a stress factor More calmness for patients and nursing More security/less unauthorized persons in the hospital Patients: Rooms with two beds Telephone consultation Fewer infections Patients focus more on themselves in some cases Digital work: More remote work possible Virtual conferences IT is getting better Improved digital working Structure and organization: Renovations Own office as a retreat Workplace is closer to the team, therefore more connection and shorter distances New constructions under required safety measures
	Work atmosphere	Togetherness and Cohesion: More conscious interaction and good behavior among each other Sense of community and joint implementation strategies Great willingness to help each other High motivation on all sides to manage the crisis Support and consideration Openness and solidarity Cohesion of the different wards Team spirit/teamwork Everyone is pulling together/mastering tasks together Some employees move closer together There is a lot of laughter Everyone in the team is equally affected Mental support Similar opinions Calmer and more relaxed work atmosphere
	Physical and chemical factors	Hygiene and infection protection: Improved hygiene standards More attention to hygiene measures Refrain from shaking hands Disinfection Increasing acceptance of the measures Knowing that individual actions do not spread the disease More routine in handling infected patients Protective measures Good infection protection More caution in certain areas (protection of others and own protection) More tests Wearing mask and protective equipment Other aspects: Air conditioning Lower noise level
Individual changes/resources/strategies	Individual resources	Friends and family: Spouse/partner Support from family, e.g., with child care Conversations Strong and positive private environment provides recovery Family as a retreat Other people with similar attitudes Understanding (in general)
		Leisure activities: Leisure time/time off in general Sports activities and relaxation (meditation, cycling, breathing exercises, outdoor sports, autogenic training) Outdoor/nature activities (walks, fresh air, hiking, forest bathing)
		Individual attitude/qualities: Confidence Positive thinking Staying calm Humor/laughing/having fun Religion/trust in God Concentration/focus on yourself Resilience Inner strength Pushing fear aside/not being afraid Good mental hygiene Many new experiences and challenges Better assessment of situations due to medical knowledge Research, dealing with the topic Self-protection and setting boundaries
Other positive changes/resources/strategies	Other resources	Being allowed to go to work instead of sitting at home to work Satisfaction with the situation at work Felt safer in the hospital than outside Distraction when working with patients Appreciate working in palliative care Being able to help others despite the pandemic Recognition

#### 3.2.1. Social relations at work

Employees said that a strong team spirit, exchange and good relations with colleagues were helpful. According to the participants, open communication among colleagues led to better cohesion. Cooperative exchange of opinions, feelings and expertise was described as useful in order to cope with stressors related to the COVID-19 pandemic. Overall, colleagues were the most frequently mentioned resource.

#### 3.2.2. Organization of work

The reduced number of examinations and operations at the beginning of the pandemic was described as helpful. Due to the low number of patients, there seemed to be more time for individual patients. The fact that fewer relatives were in the hospital and hence fewer conversations were necessary was perceived as relieving. Employees said they were able to take care of many tasks that had been previously deferred due to time constraints. They also described having less time pressure and fewer meetings and expressed that better planning was possible. The quietness apparently also brought teams together, which indicates a connection between colleagues and the work atmosphere. Some participants described this aspect as an unexpected development. Participants perceived this relief especially in the first survey stage.

Participants valued a functioning information exchange and mentioned successful communication as helpful: Regular and timely information (e.g., concerning current regulations and policies for COVID-19 infections or contact with persons infected) provided transparency and certainty in dealing with the pandemic, according to our respondents. They also perceived clarity of information and rules as well as new ways and forms of communication, such as daily information meetings where relevant changes were communicated, as helpful in handling the pandemic situation. Similar aspects were mentioned with regard to the work process: Participants appreciated clear guidelines and procedures, quick and pragmatic decisions, little confusion as well as consistent and determined structures. Further helpful aspects in this context were calmer procedures, less hecticness, growing confidence and more routine. Cooperation, e.g., collaboration and support from other departments, areas and wards or a more profound coordination with the management, was also mentioned as helpful by participants, because this seemed to give them insights into other units. However, it has to be mentioned that when participants were requested to help out in other departments, this was sometimes considered as a burden.

#### 3.2.3. Work environment

With regard to workplace and information design, participants particularly valued the adjusted visitor regulations, especially in the first survey stage. Access controls seemed to have led to more relief and safety among patients and employees. The reduction of visitor numbers was perceived as an improvement that seemed to have led to both fewer infections among patients and less stress among hospital staff. Several participants requested that these regulations should also be maintained after the pandemic.

The calmer and relaxed work atmosphere at the beginning of the pandemic was perceived as relieving. Respondents also described an improved work atmosphere with regard to collaboration in the team: Team spirit and teamwork were highlighted, and everyone seemed to be pulling in the same direction and master different tasks as a team. Some employees apparently also moved closer together. Support and consideration as well as cohesion and solidarity were described as helpful. Several respondents indicated that they had not expected these positive changes.

Improved hygiene standards and the acceptance of measures among colleagues, patients and relatives were often perceived as helpful in the context of dealing with stressors related to the pandemic. Additionally, caution, sufficient testing as well as wearing masks and protective equipment were addressed.

#### 3.2.4. Individual resources

According to our respondents, the private environment, especially joint conversations, distraction and support from partners, family and friends, e.g., with child care, often brought relief. Leisure activities, such as sports or time spent outside or in nature, were also mentioned as resources. Furthermore, participants described qualities or attitudes such as optimism, positive thinking, humor, serenity, resilience and concentration as helpful in dealing with the situation. Individual resources were important in both survey stages.

## 4. Discussion

In the present study, we examined what stressors hospital staff perceived during the pandemic and what resources were helpful for them in order to deal with stress. The answers of 303 hospital workers at two different time points in the development of the pandemic helped us identify aspects in four areas that could be reinforced for normal operation and further crises: (1) social relations at work, (2) organization of work, (3) work environment and (4) individual resources. To a large extent, our results show overlapping resources between the different occupational groups at the hospital workplace. This can be taken as an indication that there are starting points to introduce or deepen stress prevention measures in all hospital sections.

We have focused our discussion on resources that can be modified through organizational and work changes. Some resources mentioned by individual participants in our study which, to our knowledge, have not been recorded in previous studies in relation to the COVID-19 pandemic include interdisciplinary cooperation across different teams and departments as well as the gain of (medical) knowledge related to the disease. Participants in our study mentioned interdisciplinary work and cooperation among teams and departments not merely as a burden but sometimes as helpful. This stands in contrast to another study conducted in Germany where participants indicated their desire for fixed and stable teams in the first phase of the pandemic (7).

Many respondents already seemed to have great confidence in their colleagues. Our results show that the extraordinary situation of crisis brought teams and colleagues closer together, but caused conflicts as well, especially in the second survey stage. Social relations are considered one of the most important influence factors of health (35). It is already recognized that positive social relations are important for stress reduction at the hospital workplace (2, 36). Situations characterized by high stress require the mobilization of social support to prevent negative consequences of stress (20). Nevertheless, stressful situations can also erode social relations in the long run, especially when stressors are chronic (20). The increase in stressors related to social relations in the second wave of the pandemic could be an indication that this resource was already eroding.

Long working hours, time and work pressure as well as frequent overtime have been recognized as job-related stress factors (37). Temporarily lower workloads at the beginning of the pandemic, a good flow of information and successful communication and cooperation were perceived as especially helpful in a time when regulations and procedures were rapidly changing. These findings suggest that interventions to improve work organization and work environment could reduce stress of hospital staff. A rapid review on the prevention and management of psychosocial effects among healthcare workers during previous pandemics assessed clear communication and the adherence to hygiene and infection control measures as helpful strategies (38). Respondents in our study found these aspects helpful during the first two waves of the COVID-19 pandemic, which is also consistent with a qualitative study from the United States (28).

Social relations in the private environment and further individual coping strategies such as attitudes and leisure activities also played an important role in dealing with pandemic-specific stressors. This finding empirically supports the expansion of the JD-R theory, which proposes that organizational, job, home and personal demands and resources interact with each other, such as that, for example resources from either domain can buffer demands of the same or other domains and that proactive regulatory strategies of the individual can boost the positive impact of resources from different domains (24). In a crisis context, family resources become “resistance resources” that can prevent change or disruptiveness (24). Thus, the well-being of employees in times of crisis may not only be influenced by the organization or the leader, but also by families and the individuals themselves (24). In the case of care professions, individual resources have been shown to have a protective effect on workload and the risk for burnout (39). Another study reported that psychosocial support from friends and family as well as leisure time were the most frequent resources among care staff and physicians during the pandemic (7). Participants in our study also mentioned leisure time and personal contacts as important resources. Since our study included all hospital staff with patient contact, our findings suggest that employees from the functional service and secretariats also benefit from positive personal contacts and leisure activities in times of crisis.

The decrease in workload reported by our respondents during the first wave of the COVID-19 pandemic stands in contrast to reports of increased workload in hospitals in Germany (12) as well as other European countries (29) and may be specific to hospital departments not attending COVID-19 patients. Due to the temporarily lower workload at the beginning of the pandemic, it became clear that relief in this area can be perceived as especially positive and beneficial. This stands in contrast with more frequent mentions of stressors in relation to emotional demands, social relations and workload in the second survey stage. This observation is in line with one assumption of the job-demands-resources-model, namely that without sufficient opportunities for recovery, permanent stressful work demands can become stressors that can deplete the resources of employees (21).

During a crisis, employees with high job demands and low job resources are less likely to adapt to the situation and maintain well-being and performance (24). A follow-up study among Canadian nurses indicated that exhaustion due to pandemic-related stressors had not subsided a year after the pandemic, and that some were considering leaving the profession or had already done so (40). Resources become especially important in times of crisis, but they are also essential during normal operation. According to occupational psychology studies, employees who have stronger reserves of resources can handle demands resulting from stressful working conditions more effectively (20). For employees without resource reserves, on the other hand, these stress-inducing conditions can become chronic (20). Further, it has been suggested that a “recovery paradox” ensues when the need to recover from job stressors is high, while at the same time the likelihood to actually recover under these circumstances is reduced (41). Periods of high workload–e.g., later during the pandemic when operations were ramped up again–cannot be completely avoided in clinical work routine. Therefore, it is important to provide hospital employees with resources by improving the organization of work.

### 4.1. Implications

Against the background of our results, the promotion of social support and communication seems to be a promising starting point to effectively improve working conditions. Trust in the team and in other colleagues can prevent anxiety and depression among hospital staff, which is why the promotion of mutual trust through teambuilding activities is recommended (5). Conversations with colleagues and superiors have already been recognized as an especially valuable resource for stress management among care staff (39). So-called “Schwartz Rounds”, conversations among employees that focus on reflection, emotions and exchange (42), might be a helpful intervention that could be implemented even without significant structural changes. One study has shown that Schwartz Rounds can improve mutual understanding and can be beneficial for teamwork and a connection among staff (42). Yet, social relations cannot compensate stress-generating working conditions in the long run (20), which is why a parallel reduction of stress-generating demands in addition to promoting this resource is needed in order to sustainably prevent stress.

The relevance of individual resources next to organizational and job resources for hospital workers was an important result of our study. Individual resources such as leisure activities or family and friends could be strengthened by ensuring necessary regenerative breaks, e.g., through sufficient staffing and optimized duty planning. Avoiding long working hours could also protect this resource (7). The prevention of stressors becomes especially important in view of the “recovery paradox” (41), which suggests that people experiencing a high level of job stressors cannot fully profit from resources that promote recovery. At this point, the issue of staff shortage needs to be mentioned as well. The connection between difficult working conditions and staff shortages has already been examined, some studies reported that hospital managers considered high workloads a reason for absenteeism among hospital staff (36, 43). More staff in the hospital sector could possibly at least partially solve other problems described, such as additional work, compensation for absence of staff/quarantine of other employees or related dissatisfaction, which reinforce each other in a vicious circle. Improved staffing could also help ensure that employees take the necessary breaks for regeneration and thus prevent stress. However, this kind of intervention might require the involvement of further actors outside of the hospital context, as the problem of staff shortage is currently one of the big challenges in the German political landscape (44, 45).

### 4.2. Strengths and limitations

A strength of our study is that less frequently questioned occupational groups at the hospital (e.g., functional service and secretariats) also participated in the survey. Data were gathered at three different sites at two time points during the COVID-19 pandemic. An additional strength is the highly detailed coding process with multiple rounds and four researchers involved. Nevertheless, limitations must be taken into account as well. The answers to the open-ended questions were usually in the form of bullet points and often lacked context. We had no additional data that could have been considered. The use of open-ended questions in surveys has been criticized as a qualitative method because of the difficulty to interpret short answers without further context information, making it difficult to produce robust insights (31). By using written surveys, it was not possible to answer potential questions or achieve thematic saturation. Therefore, we do not exactly know whether all relevant aspects have been covered. Free text fields may not have been completed by employees who were particularly stressed or who had too much time pressure. However, this method may have allowed us to capture responses of hospital staff who may not have had the time to participate in a more time-consuming interview or focus-group study, especially during the period characterized by high workload demands. Moreover, there are only 173 employees who participated in both surveys considered by us, and we did not conduct a dropout-analysis. Statements about the development over time are therefore difficult. Even though there were two survey stages, we analyzed the results altogether. A further limitation is that data from the functional service, the secretariats and others as well as the data from physicians and the medical-technical service were each compiled in two groups for data protection reasons. This meant that we were unable to make differentiated statements with regard to the individual occupational groups, but only for the respective group in total. Finally, only hospitals that had already been involved in the SEEGEN project participated. Thus, they might have already had more resources at hand than other hospitals.

## 5. Conclusion

The resources perceived by employees in large hospitals of different ownership indicate that communication and mutual social support significantly contribute to a better coping with everyday stress and special challenges in a time of crisis. Improving workplace and communication design and reducing the amount of work were also perceived as helpful. Strengthening and reinforcing existing resources is a useful and necessary starting point for the sustainable improvement of working conditions in normal operation and in order to prepare for possible further pandemics and crisis situations. Adequate staffing of the clinics must not be disregarded in the substantial promotion of these resources.

## Data availability statement

The datasets presented in this article are not readily available because of data protection guidelines. Requests to access the datasets should be directed to KS, kira.schmidt.stiedenroth@hhu.de; LG, lisa.guthardt@hhu.de.

## Ethics statement

The studies involving humans were approved by University of Ulm: 501/18, University of Heidelberg: S-602/2019, University of Düsseldorf: 6193R. The studies were conducted in accordance with the local legislation and institutional requirements. The participants provided their written informed consent to participate in this study.

## Author contributions

KS, LG, MG, PA, and AM: Conceptualization. KS, LG, MG, and MK: Formal analysis. FJ, IM, HG, PA, AM, and SEEGEN: Funding acquisition. KS, LG, MG, and MK: Methodology. SEEGEN: Project administration: MS, RE, SEEGEN, PA, and AM: Resources. AM and PA: Supervision. KS and LG: Visualization. KS and LG: Writing - original draft. KS, LG, MG, MK, MS, RE, FJ, IM, HG, PA, and AM: Writing - review & editing.

## Funding

The authors declare financial support was received for the research, authorship, and/or publication of this article. The study was funded by the Federal Ministry of Education and Research: FKZ 01GL1752B.

## Conflict of interest

The authors declare that the research was conducted in the absence of any commercial or financial relationships that could be construed as a potential conflict of interest.

The author(s) declared that they were an editorial board member of Frontiers, at the time of submission. This had no impact on the peer review process and the final decision.

## Publisher’s note

All claims expressed in this article are solely those of the authors and do not necessarily represent those of their affiliated organizations, or those of the publisher, the editors and the reviewers. Any product that may be evaluated in this article, or claim that may be made by its manufacturer, is not guaranteed or endorsed by the publisher.
